# Bio-Inspired Aggregation Control of Carbon Nanotubes for Ultra-Strong Composites

**DOI:** 10.1038/srep11533

**Published:** 2015-06-22

**Authors:** Yue Han, Xiaohua Zhang, Xueping Yu, Jingna Zhao, Shan Li, Feng Liu, Peng Gao, Yongyi Zhang, Tong Zhao, Qingwen Li

**Affiliations:** 1 Laboratory of Advanced Polymeric Materials, Institute of Chemistry, Chinese Academy of Sciences, Zhongguancun North First Street 2, Beijing 100190, China; 2 Key Laboratory of Nano-Devices and Applications, Suzhou Institute of Nano-Tech and Nano-Bionics, Chinese Academy of Sciences, Ruoshui Road 398, Suzhou 215123, China; 3 University of Chinese Academy of Sciences, Yuquan Road 19, Beijing 100049, China; 4 Suzhou Creative Nano Carbon Co. Ltd., Ruoshui Road 398, Suzhou 215123, China

## Abstract

High performance nanocomposites require well dispersion and high alignment of the nanometer-sized components, at a high mass or volume fraction as well. However, the road towards such composite structure is severely hindered due to the easy aggregation of these nanometer-sized components. Here we demonstrate a big step to approach the ideal composite structure for carbon nanotube (CNT) where all the CNTs were highly packed, aligned, and unaggregated, with the impregnated polymers acting as interfacial adhesions and mortars to build up the composite structure. The strategy was based on a bio-inspired aggregation control to limit the CNT aggregation to be sub 20–50 nm, a dimension determined by the CNT growth. After being stretched with full structural relaxation in a multi-step way, the CNT/polymer (bismaleimide) composite yielded super-high tensile strengths up to 6.27–6.94 GPa, more than 100% higher than those of carbon fiber/epoxy composites, and toughnesses up to 117–192 MPa. We anticipate that the present study can be generalized for developing multifunctional and smart nanocomposites where all the surfaces of nanometer-sized components can take part in shear transfer of mechanical, thermal, and electrical signals.

A composite material is typically made up of two or more constituent materials with significantly different physical or chemical properties, and is also named a nanocomposite when one of the constituents has one, two, or three dimensions of less than 100 nm. To design the structure for high performance and multifunctional composites, nature has offered us with scientific and technological clues from the formation of biological composites using common organic components via the naturally mild approaches^1^. For example, super-tough spider fibers are derived from desirable organization of linear protein molecules^2^, strong hard nut skins are assembled from the mixture of cellulose and lignin molecules^3^, and wear-resistant molluscan shells are a result of biomineralization of calcium carbonates in a brick-and-mortar manner^4^. To make these natural composites mechanically strong, a homogeneous distribution of the major components such as proteins, cellulose molecules, and nanometer-sized crystals of carbonated calcium phosphates or calcium carbonates is a key structural feature^2^,^5^. Their desired orientation along with other co-existing components also sheds lights on the way to stronger man-made nanocomposites. This means, in order to fabricate high performance nanocomposites, the fraction of nanometer-sized components should be as high as possible, while the other components should act as interfacial adhesions and mortars to combine the major parts together. As a result, the interfacial contacts or bondings can be maximized to allow full utilization of the unique properties of the nanometer-sized components.

Owing to the superior mechanical properties of carbon nanotubes (CNTs), many composite structures have been proposed for pursuing a wide range of industrial applications of CNT over the past two decades^6^,^7^,^8^,^9^,^10^. As CNTs are difficult to be uniformly dispersed within polymer matrix at a high mass fraction due to their strong tendency to agglomerate^11^,^12^,^13^,^14^,^15^, it is still a challenge to fabricate CNT composites that mimic the natural ones. Fortunately, CNTs can be treated as linear macromolecules, and thus the processing on them can be dealt with in a biomimic way. To mimic the formation process of biological composites, the preformed two-dimensional (2D) CNT assemblies like sheets and films, whose thickness is within tens to hundreds of nanometer or over 1 μm, are interesting candidates^9^,^16^. By introducing thermosetting polymers like bismaleimide (BMI) into these 2D assemblies, it has been possible to synthesize CNT composites at a high CNT mass fraction^17^,^18^,^19^,^20^,^21^, whose tensile strength was even higher than that of T300 carbon fiber/epoxy composites (1.86 GPa)^22^. However, besides the high length-to-width aspect ratio and high mass fraction, a set of structural parameters are still severely required, such as a high CNT packing density and alignment, efficient matrix-to-CNT interfacial stress transfer, and, most importantly, the avoid of CNT aggregation^23^.

The necessity of aggregation control can be demonstrated by a comparison between the structures of carbon fiber reinforced polymer and CNT composites containing aggregated and unaggregated CNTs, as schematically shown in Fig. 1. The most important advantage of CNTs is the large contact area between CNTs and matrix, similar to the natural composite structures. When solid carbon fibers are replaced by the aggregated CNTs, as commonly observed in today’s CNT composites^17^,^18^,^19^,^20^,^21^, the interfacial contact area becomes much larger. The composites based on aggregated CNTs have exhibited tensile strengths ranging from 2.08 to 3.8 GPa^17^,^18^,^21^. However, the aggregation phase might act as weak parts in transferring external loads and thus hinders the further reinforcement. In an ideal structure where all the interfaces can play roles in shear load transfer, the nanometer-sized components should be uniformly distributed within the matrix and there should be no aggregation for either the matrix or the nanometer-sized structures.

Here we report a big step to approach such ideal structure where the composite structure contained highly aligned and unaggregated CNT bundles. By learning the formation process of biological composites, polymers were impregnated into CNT networks to obtain the uniform dispersion of the CNTs among the polymer matrix. As the CNTs were well covered by the polymers, sufficient stretching exercises were performed to improve the CNT alignment with maintaining the CNT aggregation level below 20–50 nm, and to increase the mass density as well. The new CNT composites exhibited ultra-high and stable tensile strengths up to 6.27–6.94 GPa and toughnesses up to 117–192 MPa, corresponding to the energies absorbed before rupturing of 75–124 J g^-1^ by considering the mass density of ~1.55 g cm^−3^. Such tensile strengths have been more than 100% higher than those of carbon fiber/epoxy composites. The processing method is supposed to be generalized for developing multifunctional and smart nanocomposites where all the surfaces of nanometer-sized components can take part in shear transfer of mechanical, thermal, and electrical signals.

## Results

### Entangled CNT network

The CNT aggregation arises from van der Waals (vdW) attraction and can be enhanced in wet environment. The situation becomes very severe in the layer-by-layer stacking of aligned CNT sheets with the aid of solution spray to obtain high performance CNT films^19^,^20^,^21^, where the CNTs (or more commonly, small-sized CNT bundles) usually aggregate first into large-sized bundles and then are surrounded by polymer matrix, as discussed below. Instead, preformed CNT networks can be the optimal raw materials^17^,^18^.

The networked CNTs were synthesized by using an injection chemical vapor deposition (CVD) method^24^, where a mist of ethanol, ferrocene, and thiophene was injected into a heated gas flow reactor (see Supplementary Information). The grown CNTs cross-linked with each other, formed a sock-like aerogel in the gas flow, and were blown out with the carrier gas, a mixture of Ar and H_2_. By continuously winding the CNT aerogel on a roller with the aid of ethanol densification, CNT films with a thickness of 10–30 μm were obtained.

Scanning electron microscopy (SEM) and transmission electron microscopy (TEM) have shown that the basic structural units of the as-produced CNT films were small-sized bundles (Fig. 2a), with a diameter of 40–50 nm and containing about 50 CNTs (Fig. 2b). The CNTs in a bundle usually grew out of the same iron catalyst nanoparticle and thus became always bundled during the growth. Under the gas flow, the bundles contacted with each other and finally formed an entangled assembly structure. The CNTs were mainly double-walled and had a diameter of 1–2 nm (Fig. 2c), and were confirmed with Raman spectroscopy (see Supplementary Information, Figure S1). The as-produced CNT films had a high level of crystallinity as reflected by the high G to D-band Raman intensity ratio, and the CNT mass fraction was over 90% (see Supplementary Information, Figure S1). Furthermore, nitrogen adsorption/desorption measurement revealed a specific surface area of 119 m g^−2^.

### Impregnation without introducing aggregation

It is very important to find that the CNT entanglement was not altered after liquid densification. After further being densified with acetone, the pore sizes of CNT films decreased from >500 nm (Fig. 2a) to ~100–200 nm (see Supplementary Information, Figure S2), while the feature of random distribution and unaggregation did not change. Such process is reminiscent of the formation process of biological composites, where the matrix co-exists with and disperses the major components as they are simultaneously grown from stem cells. Their fractions are always optimized during the growth to allow the maximized interfacial stress transfer^23^. Thus the processing sequence was modified to introduce impregnation of polymer solution prior to any other processing that might damage the network, to avoid CNT aggregation. (Notice that, besides the severe CNT aggregation^19^,^20^,^21^, the pre-aligned CNT sheets drawn out of CNT arrays^25^ are not favorite also because that it is difficult to wet them as they are mechanically very weak.)

By appropriately using acetone as solvent to dissolve thermosetting polymers or their resins, like BMI resins, the polymers can efficiently cover all the CNT surfaces. Excitingly, neither the CNTs nor the resins formed aggregated phases; there did not exist a region filled with only CNTs or BMI resins above a size scale of 50–100 nm (see below the detailed characterization). The entanglement played the key role, because the capillary force due to solvent evaporation and the vdW interaction between CNT and resin could densify the assembly by drawing the CNTs closer, but these interactions were not strong enough to break the network cross-links and thus to aggregate the CNTs.

### High alignment and ultra-high strengths

Stretching should be provided to re-assemble the network and align CNTs. This requires the samples to possess high plasticity. The raw films could be stretched by 10–15% in length and owing to the improved alignment their tensile strength increased from 180–198 to 500–600 MPa (see Supplementary Information, Figure S3). For the “wet” films where 1 wt% BMI resin/acetone solutions were impregnated to reach a CNT-to-resin mass ratio of 7:3 or 8:2, the unstretched films became more plastic and fractured above a strain of 20–25%, corresponding to higher processability (see Supplementary Information, Figure S4). This means that the impregnation prior to stretching also resulted in improved processability.

If hot-pressing was applied on the unstretched films to cure resins, the CNT/BMI films finally exhibited a tensile strength just of 478–501 MPa and strain at break of 10–12.2%. On the contrast, by first stretching the “wet” film by 20% and then curing the film, the tensile strength and strain at break became 1.74–1.92 GPa and 3.4–5.2%, respectively (see Supplementary Information, Figure S6).

Nevertheless, ~2 GPa was not the up limit. By further modifying the stretching method to a multi-step way (Fig. 3a), the “wet” films could be stretched by 27–34%. The total stretching process was carried out in multiple steps. In each step 3% additional stretching according to the immediate film length was applied and then 5–10 minutes were used to relax the films. The total stretching magnitude, for example, was 1.03^8^ − 1 = 0.267 or 1.03^10^ − 1 = 0.344 for 8 or 10 steps, respectively. The multi-step method fully aligned the CNTs and improved packing density during the hot-pressing (owing to the decreased level of CNT waviness and less unstretched network connections). At this stage, the basic structural units (the small-sized CNT bundles) were well surrounded by the BMI resin molecules and maintained unaggregated phases. After being cured, the CNT/BMI composite films stably exhibited an extremely high tensile strength up to 4.5–6.94 GPa (Table 1, and also see Supplementary Information, Figure S7), depending on the CNT-to-resin mass ratio and the total stretching magnitude. At the same time, the elastic modulus was up to 232–315 GPa and the strain at break became 2.7–4.5%. Figure 3b shows the typical stress-strain curves for various CNT/BMI composite films and Fig. 3c provides the comparison with carbon fiber/epoxy composites.

### Effect of low-softening-point resins

The high mechanical performance also came from the low-softening-point (<60 °C) BMI resins (l-BMI) which were traditional BMI monomers modified with diallyl bisphenol A (DBA)^26^. As a comparison, the same DBA modification was applied on BMI monomers with larger molecular weights and higher molecular rigidity, to synthesize BMI resins with higher softening point of >80 °C (h-BMI). The low softening point resulted in a soft and viscous state even at room temperature, like soft wax, and thus the CNT film impregnated with BMI resins was called as a “wet” film. The less-“wet” CNT/h-BMI films (CNT-to-resin mass ratio 7:3) could be only stretched directly by 16% while the “wet” CNT/l-BMI films (7:3) were by >20% (see Supplementary Information, Figure S5). Of great importance, the “wet” feature also allowed structural relaxation as sufficient as possible during the multi-step stretching process. Notice that, the 16%-stretched CNT/h-BMI composite films exhibited a tensile strength of ~1.5 GPa, as comparable to the CNT/l-BMI composites (see Supplementary Information, Figure S6), indicating that the major difference of the resins was the plasticizing ability rather than the strengthening ability.

### Structural characterization

**Figure 1:**
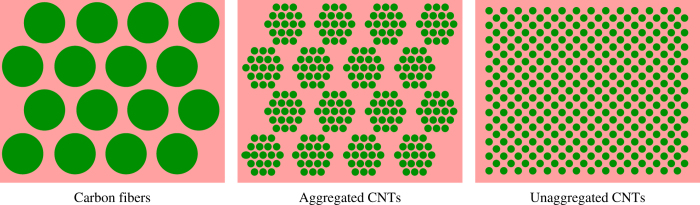
Schematics of carbon fiber reinforced polymer, composite structure with aggregated CNTs, and the ideal structure containing unaggregated CNTs, respectively.

**Figure 2:**
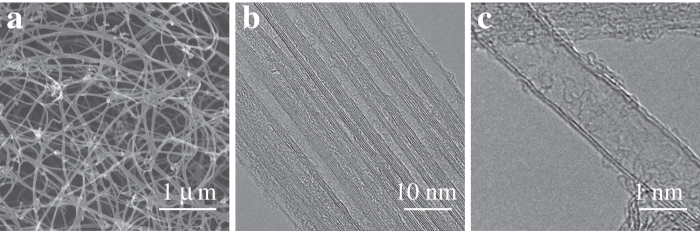
Assembly structure of as-produced CNT films. (**a**) CNT bundles contacted with each other and formed a network. (**b**) The bundle size was ~50 nm in width. (**c**) Double-walled CNTs were the major growth output.

**Figure 3:**
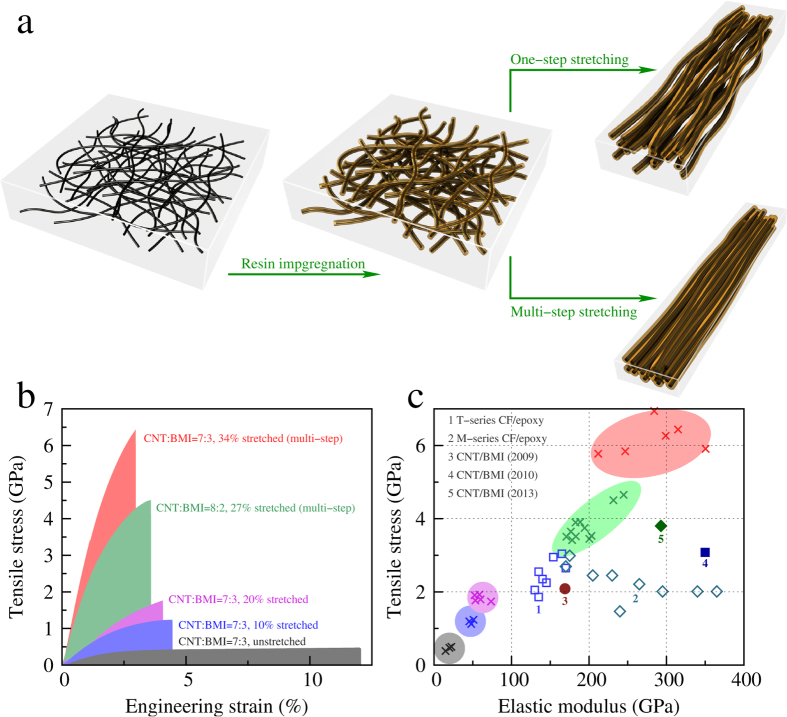
Schematic of stretching methods and mechanical properties of CNT/BMI composite films. (**a**) The polymer solution is impregnated into CNT films prior to the stretching process. The processing can be performed in one-step and multi-step ways, which result in different levels of CNT alignment and densification. (**b**) Typical stress-strain curves for CNT/BMI composite films prepared by different stretching methods. (**c**) Comparison of tensile strength and modulus of different CNT/BMI composite films, labelled by × with the same color shown in (**b**) to the T- and M-series carbon fiber/epoxy composites and recently reported high performance CNT/BMI composite films^17^,^18^,^21^.

**Figure 4:**
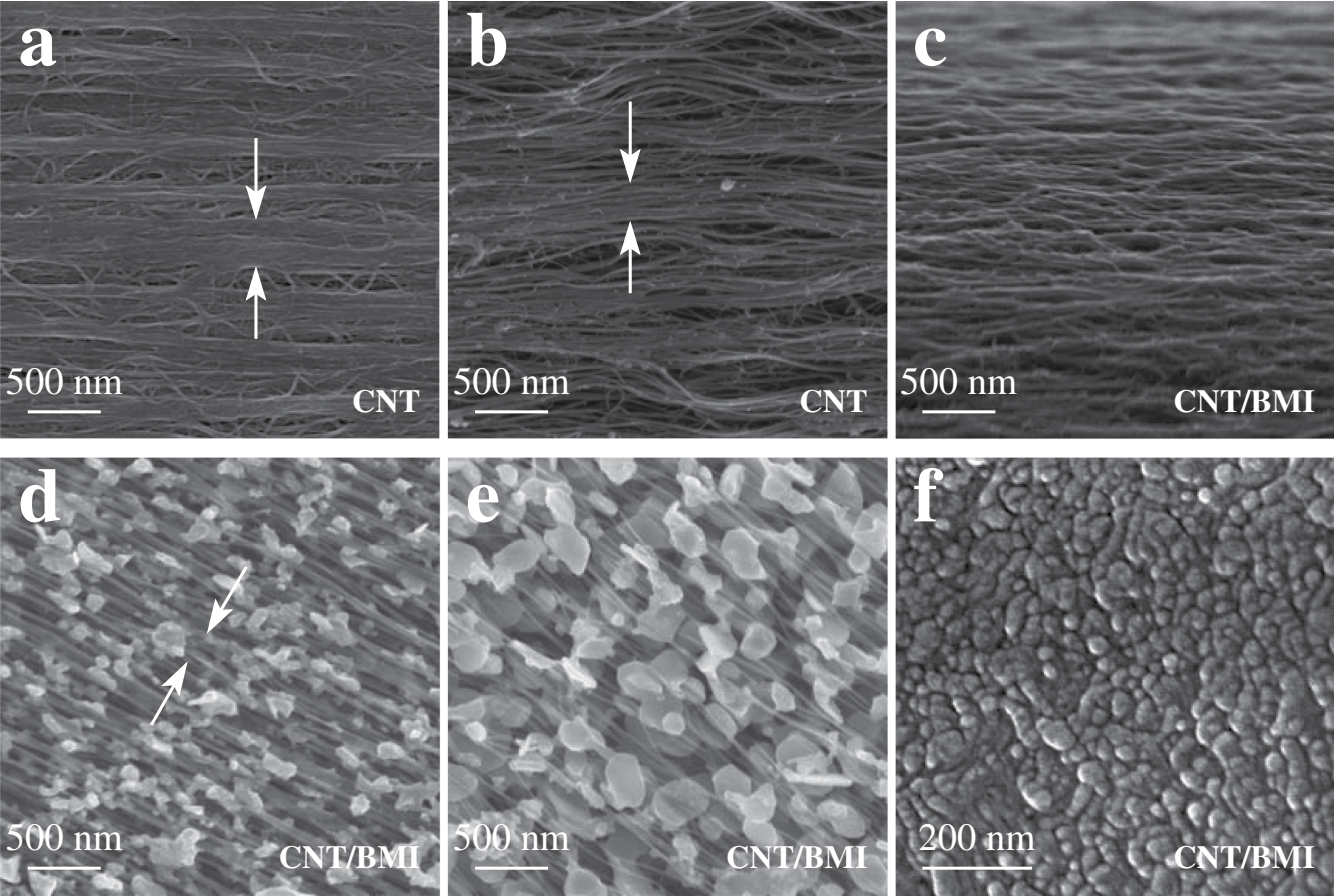
Comparison of CNT assembly structure for different films. (**a**) CNT aggregation in the layer-by-layer stacking of array-drawn CNT sheets. (**b**) CNT aggregation in the stretched dry films composed by entangled CNTs. (**c**) The small-sized CNT bundles did not aggregate but were surrounded by BMI polymers and uniformly distributed. (**d,e**) 750 °C treated CNT/BMI composite films where the stretching of 21% and 27% was performed before and after resin impregnation, respectively. (**f**) Cross section of the optimal CNT/BMI composite structure by using focused ion beam treatment.

Based on these advantages, including the unaggregation and high alignment, we have made a big step to realize the ideal composite structure. The tensile strength of >6 GPa is obviously much larger than those of carbon fiber/epoxy composites (Fig. 3), in good agreement with their different composite structures (Fig. 1). To show how much the present structure had approached the ideal one, comparison was performed between the layer-by-layer stacked array-drawn CNT sheets, highly stretched film with entangled CNTs, and the optimal and ultra-strong CNT/BMI composite films (Fig. 4a–c). In the first two cases, CNT aggregation was widely observed in a scale of hundreds of nanometer (Fig. 4a,b), while the small-sized CNT bundles did not aggregate but were surrounded and adhered with each other by BMI polymers in the optimal composite structure (Fig. 4c).

Thermal treatment at 750 °C in Ar for 1.5 h was performed on the CNT/BMI composite films to decompose BMI polymers. After the decomposition, the remaining BMI polymers formed flake-like particles, and thus exposed the CNTs which might maintain their aggregation level. Therefore this method can serve as evidences for CNT aggregation and unaggregation. Two CNT/BMI composite films were tested, where the stretching process was performed before and after the impregnation of BMI resins, respectively. As shown in Fig. 4d,e, there was clearly no aggregation of CNT bundles by using our new processing method.

It was possible to observe directly the cross section of the optimal structure by using focused ion beam treatment (Fig. 4f). Although it was difficult to distinguish individual CNTs, the small-sized CNT bundles were pictured perpendicular to the cross section and polymer matrix surrounded all their surfaces. Such homogeneity maximized interface contacts and thus provided the most efficient CNT-to-polymer stress transfer. By considering the fact that the bundling within a dimension of 20–50 nm was only determined during the growth, there is still a last step to obtain the ideal structure where individual CNTs are aligned, highly packed, and unaggregated.

Furthermore, it is no doubt that the stretching treatment improved the CNT alignment, nevertheless, quantitative characterization of alignment is still of great interest. In the present study, the characterization was represented by the Herman’s orientation factor (HOF) which has been used to study the alignment level for CNT arrays^27^. HOF takes the value 1 for a system with full alignment and zero for completely nonoriented structures. The HOF of the original film was only 0.209, well reflecting the random CNT distribution. After being stretched by about 20% and 34%, the HOF increased remarkably up to 0.632 and 0.816, respectively. (The calculation method and the detailed results are provided in Supplementary Information.)

### Specific strengths

Another way to describe the tensile property is specific strength, also known as the strength-to-weight ratio. In this way it not necessary to know the film thickness. For the films multi-step stretched by ~25% in length, the total mass for a 2 cm × 1 cm sample was 1.15 mg, corresponding to an area density of 0.58 μg cm^−2^. The fracture force per sample width was about 16.5 N mm^-1^ in average (see Supplementary Information, Figure S12), and thus the specific strength (by dividing the force per width by the area density) was ~2.87 N tex^−1^. When the stretching magnitude was improved to 34%, the fracture force per width was ~19.5 N mm^-1^ (see Supplementary Information, Figure S12), the area density decreased slightly to 0.46 mg cm^−2^, and the specific strength was ~4.24 N tex^−1^.

The volumetric mass density for the 34% stretched film was measured to be ~1.55 g cm^−3^ according to Archimedes’ principle, where the film became suspended within a mixed solution containing dichloromethane (CH_2_Cl_2_) and diiodomethane (CH_2_I_2_) with a volume ratio of 13:2. Therefore, the engineering strength (product of specific strength and volumetric density) should be about 6.57 GPa.

### Damping properties

**Figure 5:**
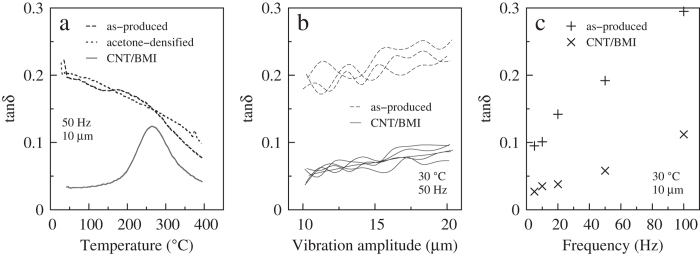
Loss factor of various films as functions of temperature (**a**) vibration amplitude (**b**) and frequency (**c**).

The entanglement of CNTs resulted in high damping performance for the as-produced dry films (Fig. 5). The loss factor tan*δ* at 50 Hz was nearly 0.2 at room temperature, decreased gradually to 0.1 as being heated up to 400 °C (Fig. 5a), and linearly increased with vibration amplitude (Fig. 5b) and frequency (Fig. 5c). The mechanism for the high damping performance was suggested to be the sliding and de-bonding between CNT bundles.

For the composite structures, these energy-cost phenomenon nearly disappeared as the BMI polymers made all the bundles adhered to each other and there no longer existed the so-called “interfaces” between different aggregation phases. At room temperature, the loss factor was even smaller than 0.05 for low-frequency vibrations (Fig. 5c). However, due to the glass transition of polymer, the loss factor increased remarkably above 200 °C.

The high loss factor of the as-produced films (comparable to rubber) to makes it possible to develop new-type high damping materials, based on the arrangement-induced viscoelastic behavior^28^, while the CNT/BMI composite films can be developed as superior structural materials to be used in aerospace, automotive, and other transportation industries.

### Electrical properties

The ability to conduct electricity of a thin film is usually characterized by sheet resistance or square resistance, in units of “ohms per square”. The square resistance of the as-produced film was 1.194 Ω sq^−1^, by using the four-point probe method. After being impregnated with BMI resins, the resistance decreased to 0.926 Ω sq^−1^, owing to the densification effect. After being stretched, the CNT network was aligned and the connections between CNT bundles were separated by BMI resins. As a result, the resistance increased to 1.461 Ω sq^−1^. The curing process finally fixed the composite structure where neither CNTs nor polymers aggregated, and the resistance further increased to 1.931 Ω sq^−1^. By considering the final film thickness of ~3 μm, the electrical conductivity was ~1.7 × 10^5^ S m^−1^, about 0.3% or 12% of copper’s or stainless steel’s electrical conductivity.

In summary, based on the unique properties of raw materials of CNTs and resins (entanglement, unaggregation, high plasticity, and low softening point) and the multi-step stretching method, we have been able to obtain a magic composite structure where neither CNTs nor polymers formed aggregated phases, a big step to approach the ideal composite structure that can fully utilize all the CNT surfaces in load transferring. The highest tensile strength was up to 6.94 GPa (or about 4.24 N tex^−1^), much higher than the strength of carbon fiber reinforced polymers. The CNT/BMI composite films also exhibited high ability to conduct electricity. As in such composite structure nearly all the surfaces of nanometer-sized components can be used, based on the bio-inspired aggregation control, we anticipate that the present fabrication method can be generalized for developing multifunctional and smart nanocomposites.

## Methods

The CNTs were mainly double-walled and were synthesized with an injection chemical vapor deposition method^24^. The grown CNTs formed a sock-like aerogel and were winded on a roller with the aid of ethanol densification to obtain 2D CNT films. The as-produced CNT films were impregnated by BMI resin/acetone solutions with designed CNT-to-resin mass ratios. The optimal CNT-to-resin mass ratio was about 7:3. Then the resin-impregnated films were stretched by more than 30% in length, in a multi-step way where sufficient structural relaxation was allowed after every step. The stretched films were cured according to the designed profile, namely, 140 °C for 0.5 h, 170 °C for 3 h, 220 °C for 2 h, and 250 °C for 3 h, with a pressure of 6–8 MPa.

Tensile tests were performed on an Instron 3365 Universal Test Machine (Instron Corp., Norwood, USA) at a strain rate of 0.5 mm min^−1^. The film samples were cut into 2.5–3 cm × 0.5–2 mm pieces, and the gauge length was larger than 10 mm. Some tensile tests to show the processability were also performed on the “wet” films with a larger width of 5–10 mm.

Dynamic mechanical analysis was carried out with a Netzsch DMA 242E Analyzer (Netzsch-Gerätebau GmbH, Selb, Germany). Temperature-dependent loss factor (tan*δ*) was measured in the temperature range of ~30–400 °C at a heating rate of 10 °C min^−1^ and a vibration amplitude of 10 μm. Another scanning mode was performed where the vibration amplitude was tuned from 10 to 20 μm, at room temperature. The allowed vibration frequencies included 5, 10, 20, 50, and 100 Hz. The sample’s gauge length was 6 mm, corresponding to the dynamic vibration strain was 0.17%–0.33%.

## Additional Information

**How to cite this article**: Han, Y. *et al*. Bio-Inspired Aggregation Control of Carbon Nanotubes for Ultra-Strong Composites. *Sci. Rep*. **5**, 11533; doi: 10.1038/srep11533 (2015).

## Supplementary Material

Supplementary Information

## Figures and Tables

**Table 1:** Mechanical properties of CNT/BMI composite films.

No.	Strength (GPa)	Modulus (GPa)	Toughness (MPa)	Strain at break (%)
CNT-to-resin ratio 7:3, multi-step stretched by 34%				
1	6.940	284.2	191.7	4.33
2	6.438	314.9	114.6	2.97
3	6.265	299.0	117.1	3.10
4	5.907	350.6	82.1	2.34
5	5.842	246.8	104.3	3.17
6	5.773	211.9	163.2	4.49
CNT-to-resin ratio 7:3, multi-step stretched by 25%				
1	6.309	148.7	197.9	5.41
2	5.781	127.9	173.3	5.41
3	5.130	168.0	187.4	5.47
4	4.467	111.3	127.1	5.02
5	4.266	146.5	123.8	4.51
6	3.826	153.6	100.4	3.95
CNT-to-resin ratio 8:2, multi-step stretched by 27%				
1	4.651	244.7	83.0	2.87
2	4.505	231.5	105.9	3.58
3	3.748	194.3	89.8	3.58
4	3.646	176.6	92.9	3.84
5	3.515	183.0	83.3	3.60
6	3.506	170.9	78.3	3.38
