# Supplementary material for: Bio-Inspired Aggregation Control of Carbon Nanotubes for Ultra-Strong Composites
Source: Sci Rep. 2015 Jun 22;5:11533. doi: 10.1038/srep11533 (PMC4476433; doi:10.1038/srep11533)
Supplement: Supplementary Information [file srep11533-s1.pdf]

Supplementary Information

Bio-Inspired Aggregation Control of Carbon Nanotubes for Ultra-Strong Composites

Yue Han,[1,3] Xiaohua Zhang,[2] Xueping Yu,[2] Jingna Zhao,[2] Shan Li,[1,3] Feng Liu,[1] Peng Gao,[4] Yongyi Zhang,[2] Tong Zhao,[1] Qingwen Li[2]

[1]Laboratory of Advanced Polymeric Materials, Institute of Chemistry, Chinese Academy of Sciences, Zhongguancun North First Street 2, Beijing 100190, China

[2]Key Laboratory of Nano-Devices and Applications, Suzhou Institute of Nano-Tech and Nano-Bionics, Chinese Academy of Sciences, Ruoshui Road 398, Suzhou 215123, China

[3]University of Chinese Academy of Sciences, Yuquan Road 19, Beijing 100049, China

[4]Suzhou Creative Nano Carbon Co. Ltd., Ruoshui Road 398, Suzhou 215123, China

E-mail: xhzhang2009@sinano.ac.cn; tzhao@iccas.ac.cn; qwli2007@sinano.ac.cn

1. Preparation of entangled carbon nanotube films

The CNTs were synthesized by using an injection chemical vapor deposition (CVD) method[1], where a mist of ethanol, ferrocene (2 wt%), and thiophene (1 vol%) was injected at a rate of 20–30 ml/h into a heated gas flow reactor (diameter 80 mm). A gas mixture of Ar (3500 sccm) and H_2 (4250 sccm) were also injected into the reactor tube as a carrier gas. The temperature in reaction region was set to 1300 °C. The grown CNTs formed a sock-like aerogel in the gas flow and were blown out with the carrier gas. The CNT aerogel was winded on a roller with the aid of liquid densification (ethanol was used here). By controlling the winding number, CNT films with a thickness ranging from 10–30 μm were finally obtained.

The basic structural units of the as-produced CNT films were small-sized CNT bundles, which usually had a diameter of 40–50 nm and contained about 50 CNTs. Once the ferrocene concentration or the injection rate is increased, larger-sized bundles can be obtained. By controlling the growth parameters, such as the concentration of ferrocene and growth temperature, it was possible to change the number of CNTs in a bundle and number of walls of individual CNT.

Raman spectra of the CNTs showed a high G to D-band intensity ratio (>5), corresponding to a high level of crystallinity (Figure S1a). The Raman signals of radial breathing modes (RBMs) also

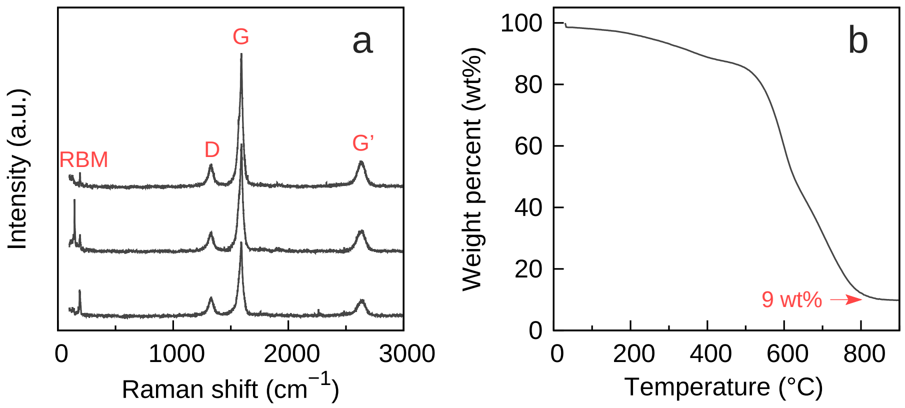

Figure S1: Characterization of as-produced CNT films. (a) Raman spectra obtained at different positions of one film. (b) Thermal gravimetric analysis indicated that the CNT content was more than 91 wt% by considering the oxidation of iron.

indicated that there existed a large number of double-walled CNTs. Further, thermal gravimetric analysis showed that there was about 9 wt% mass left for the as-produced CNT films after being heated up to 800 °C in air (Figure S1b).

2. Entanglement after liquid treatment

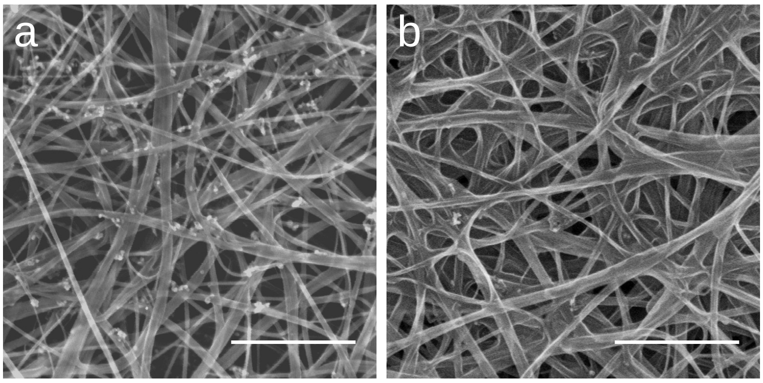

Figure S2: Acetone-densified assembly networks of CNT bundles. Scale bars are 500 nm.

As discussed in the main text, the entanglement was not altered after liquid densification. Figure S2 shows two acetone-densified CNT networks. As compared to Figure 2a (in the main article), it is clear that the pore size had decreased from >500 nm to ∼100–200 nm, while the feature of random distribution and aggregation structure was maintained. The densification mainly took place along the direction perpendicular to the film surface, that is, different CNT layers (due to the winding

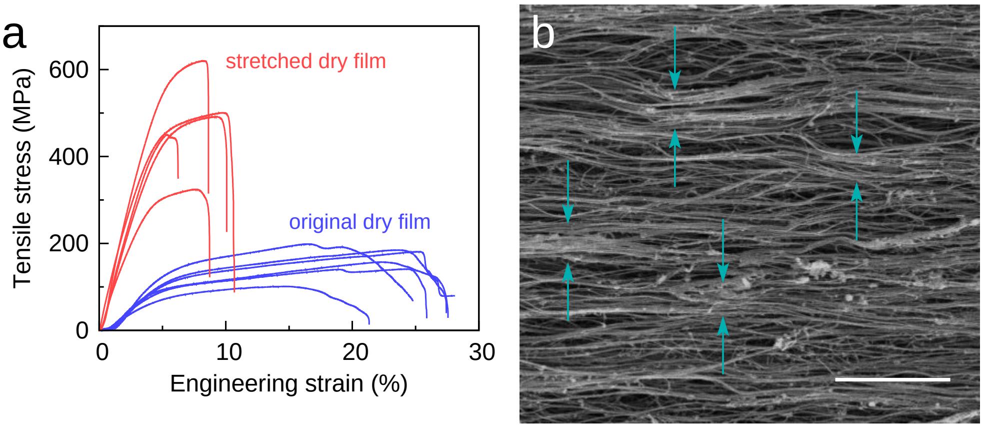

Figure S3: Mechanical properties (a) and CNT alignment (b) of directly stretched films. Scale bar is 2 μm.

process) were drawn closer, while the shrinkage of film width and length was less than 2–5% (but measurable). Therefore, it became possible to introduce resin molecules into the CNT films without aggregating the CNT bundles.

3. Mechanical properties of dry films

As the bismaleimide (BMI) resins had a softening point smaller than 60 °C and demonstrated a viscous liquid property, we call the resin-impregnated CNT films as "wet" films while the pure CNT films as dry ones. The tensile strength, strain at break, and toughness (the area covered under stress-strain curve) of the as-produced dry films were up to 180–198 MPa, 20–26%, and 32–37 MPa, respectively (Figure S3a). Stretching the dry films to align CNTs is the simplest method to improve the mechanical properties. By stretching directly by 20% within one minute and then maintaining the stretching to relax for 10–30 min, the films exhibited improved strengths and toughness, up to 492–620 MPa and 35–40 MPa, respectively. In the stretched film, CNTs became much more aligned (Figure S3b), making the modulus larger than 12 GPa.

It is important to notice that, during the stretching process the CNTs not only became aligned, but also aggregated to form large-size bundles (as labelled by arrows in Figure S3b). The aggregation can benefit the load transfer between CNTs in the stretched dry films, however, might become weak part in composite films by impregnating BMI resins and then curing them. This is because the aggregated CNTs transfer loads just depending on the intertube sliding friction while the BMI network can increase significantly the interfacial interactions with CNT surfaces and connect non-

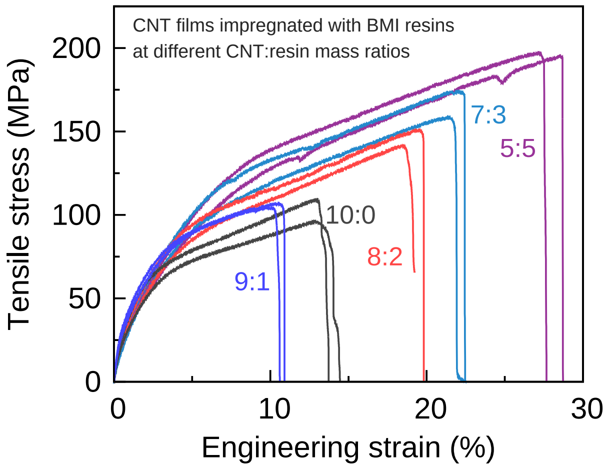

Figure S4: Tensile characterization of CNT films impregnated with different amount of BMI resins.

neighboring CNTs, resulting in much more efficient load transfer within polymer-bridged CNTs [2–5]. This means, getting rid of CNT aggregation has become a challenge as important as improving interfacial interaction and CNT alignment. To further confirm the effect of aggregation, BMI resins were impregnated into the stretched dry films. As the aggregation existed, such CNT/BMI composite film exhibited a tensile strength up to only 585–801 MPa, after being cured.

4. High plasticity of "wet" films

To avoid CNT aggregation, the as-produced CNT films were impregnated with 1 wt% BMI resin/acetone solutions prior to the stretching process. By controlling the total volume of solution, "wet" films at different CNT-to-resin mass ratios were produced, namely 9:1, 8:2, 7:3, and 5:5 (similarly, the ratio for dry films was 10:0). The "wet" films had different strain at break at different mass ratios (Figure S4). When a small amount of resins were impregnated, the resin molecules could not sufficiently cover the CNT surfaces and thus caused significant inhomogeneity within the film. Therefore the film became easier to fracture. When more resins were used, the film became more and more plastic and even can be stretched by $\sim$30%. This means that the wet environment gives the film enhanced processability.

Notice that the 10:0 sample had a different strain at break from the tensile tests shown in Figure S3a. This is because the widths of the test samples were 10 and 2 mm for the samples in Figure S4 and S3, respectively. As compared to small samples, assembly defects (in the as-produced CNT

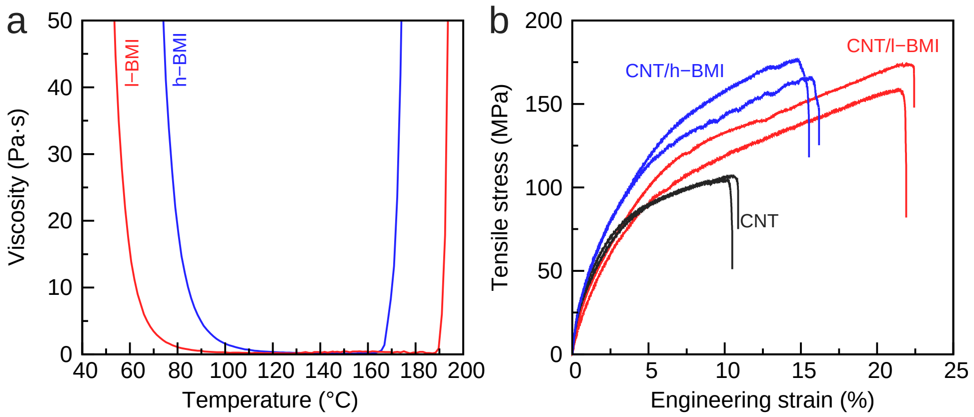

Figure S5: Viscosity of l-BMI and h-BMI resins (a) and their influences on processability of CNT/BMI composite "wet" films (b).

network) make the fracture easier for large ones. In fact, the tensile results of large samples reflected the real processability, as the resin impregnation and stretching processes were performed on CNT films with a width of 1 cm.

5. Low-softening-point bismaleimide resins

The low-softening-point BMI (l-BMI) resins were synthesized by modifying traditional BMI monomers with diallyl bisphenol A (DBA) [6]. Besides the DBA modification, the original molecular structure of BMI monomers also determines remarkably on the softening dynamics. As a comparison study, the DBA-modified resins with larger molecular weight of monomers, higher molecular rigidity, and thus higher softening point, the h-BMI resins, were also used to show their influences on the processability of CNT/BMI composite films. Rheological measurement, the viscosity-temperature relationship, reveals that the viscosity of l-BMI decreased to below 20 Pa s at ∼58 °C while such temperature was ∼80 °C for h-BMI (Figure S5a). By impregnating them into CNT films at the same mass ratio of 7:3, the tensile measurements showed different increase in strain at break. The CNT/h-BMI "wet" film fractured at ∼15% while the CNT/l-BMI at more than 22% (Figure S5b, sample width 10 mm).

6. One-step and multi-step stretching methods

The stretching methods are schematically shown in Figure 3 in the main text. Here we provide more details about the difference between the one-step and multi-step treatments.

The as-produced CNT films are first impregnated by BMI resin/acetone solution. As acetone has

a high infiltration ability into CNT assemblies, the impregnation can easily make all the CNT bundles surrounded by the resin molecules and avoid the aggregation of CNTs. “Wet” films containing a designed CNT-to-resin mass ratio are prepared by using the low-softening-point resins. These “wet” films can be directly stretched by $\sim$20% in a one-step way. The one-step stretching can effectively align the CNTs, however, as the lack of relaxation during the stretching, the entangled or cross-linked segments can not be fully aligned and thus hinder the densification process.

A multi-step stretching method is used to introduce the relaxation process. In this way, the “wet” film is always stretched slightly in each step, by 2–3%, and there are 5–10 minutes to relax the film before the next stretching. If the stretching magnitude ξ depends on the current film length, after n steps, the total stretching magnitude can be calculated by $(1+\xi)^n - 1$. (The stretching magnitude can be confirmed by the final length increase as compared to the original length.) If the stretching length x is always fixed, the total stretching magnitude is then nx/L_0 where L_0 is the original film length.

The relaxation process plays important roles in optimizing CNT alignment and level of densification for the “wet” films, which can be represented by the mechanical properties of the cured samples and the mass density as well.

7. Mechanical properties of one-step stretched films after curing at mass ratio of 7:3

The “wet” films were cured by a hot-pressing process with a pressure of 6–8 MPa. The curing profile was 140 °C for 0.5 h, 170 °C for 3 h, 220 °C for 2 h, and 250 °C for 3 h. The introduction of 140 °C treatment was found to be also important to liquidize the resin molecules and further improved homogeneity in the composite structure.

The one-step stretching was applied by 10% and 20%, respectively, on “wet” films with the CNT-to-resin mass ratio of 7:3. After being cured, the two stretched composite films exhibited tensile strengths of 1.12–1.24 GPa and 1.74–1.92 GPa, respectively, while the unstretched composite films had strengths less than 500 MPa (Figure S6). This can be ascribed to the insufficient alignment of CNTs. Furthermore, as compared to the multi-step stretching, the lack of relaxation process resulted in inhomogeneity in the composite structure. For example, when the “wet” films were stretched by 19% in the multi-step way, there should not be a significant difference in alignment as compared to the 20% one-step stretched films. However, the inhomogeneity in one-step stretched

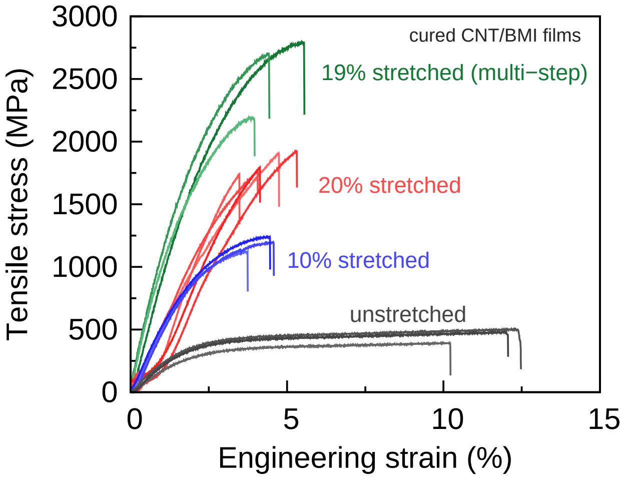

Figure S6: Comparison of tensile properties between different CNT/BMI films which include the unstretched, one-step stretched (by 10% and 20%), and multi-step stretched (by 19%) films. The CNT-to-resin mass ratio was 7:3.

films reduced the efficiency of load transfer between CNT bundles and thus hindered the increase in elastic modulus. As a result, their tensile strengths of 2.19–2.79 GPa were much larger, at least by 450–870 MPa.

8. Effect of multi-step stretching

In the multi-step stretching, we set $\xi = 3\%$ and the relaxation time to be 10 min, and thus the total stretching magnitude was $1.03^n - 1$. The maximum number of steps differed from sample to sample. For the 7:3 "wet" film $n \leqslant 10$ while for the 8:2 film $n \leqslant 8$. We also tried to stretch the film by a fixed length of 1 mm in each step (the original length and width was about 35 mm and 10 mm, respectively). However, we did not observe any difference between these two treatments. To further increase the maximum stretching magnitude, studies should be performed on the CVD process where the CNT structure and formation of CNT network are determined.

Figure S7 shows the stress-strain curves of cured CNT/BMI films after being multi-step stretched. The highest performance was found for the 7:3 films being stretched by 34% ($1.03^{10} - 1$). The tensile strengths and moduli (both in units of GPa) of these tests were 6.940/284.2, 6.438/314.9, 6.265/299.0, 5.907/350.6, 5.842/246.8, and 5.773/211.9, respectively. Even just being stretched up to 25%, the tensile strength was still found to range from 3.83 to 6.31 GPa, higher than traditional

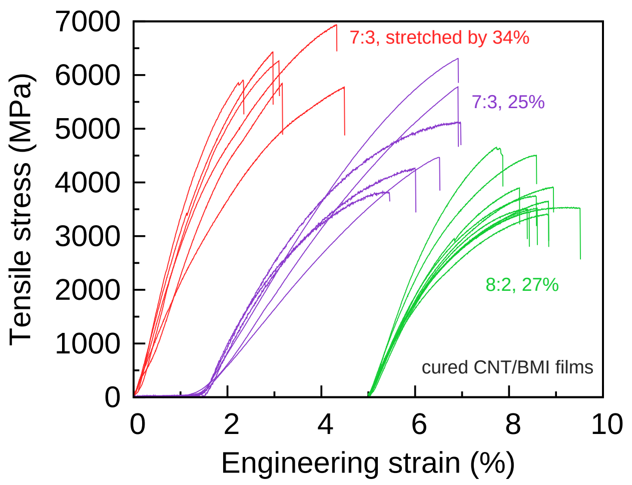

Figure S7: Stress-stain curves for multi-step stretched CNT/BMI films where the stretching magnitudes were larger than 25%. Some plots are shifted for a better comparison.

carbon fiber/epoxy composites and recently reported high performance CNT/BMI composites [2,3,5].

As compared to the 7:3 mass ratio, the 8:2 ratio resulted in a maximum stretching magnitude of ∼27% and final tensile strengths ranging from 3.42 to 4.65 GPa. This means that for high performance composites based on polymer impregnation into CNT assemblies, the polymer content should be around 30 wt%. When more BMI were introduced into the film, like mass ratios of 6:4 and 5:5, the BMI aggregation appeared and became structural defects to make the film more brittle than the 7:3 or 8:2 ones.

9. Herman's orientation factor

Herman's orientation factor (HOF) is a commonly used parameter to characterize orientation and has been successfully used for determining the alignment level for CNT arrays [7]. It takes the value 1 for a system with perfect orientation parallel to a reference direction, and zero for completely nonoriented samples. The calculation of HOF is based on the intensity profile I of orientation angle ϕ between the structural unit vector and the reference direction, according to its definition

$$\mathrm{HOF} \equiv \frac{1}{2}\left(3\left\langle\cos^2\phi\right\rangle - 1\right), \tag{1}$$

where

$$\left\langle\cos^2\phi\right\rangle = \frac{\int_0^{\pi/2} I(\phi)\cos^2\phi\sin\phi\, d\phi}{\int_0^{\pi/2} I(\phi)\sin\phi\, d\phi}. \tag{2}$$

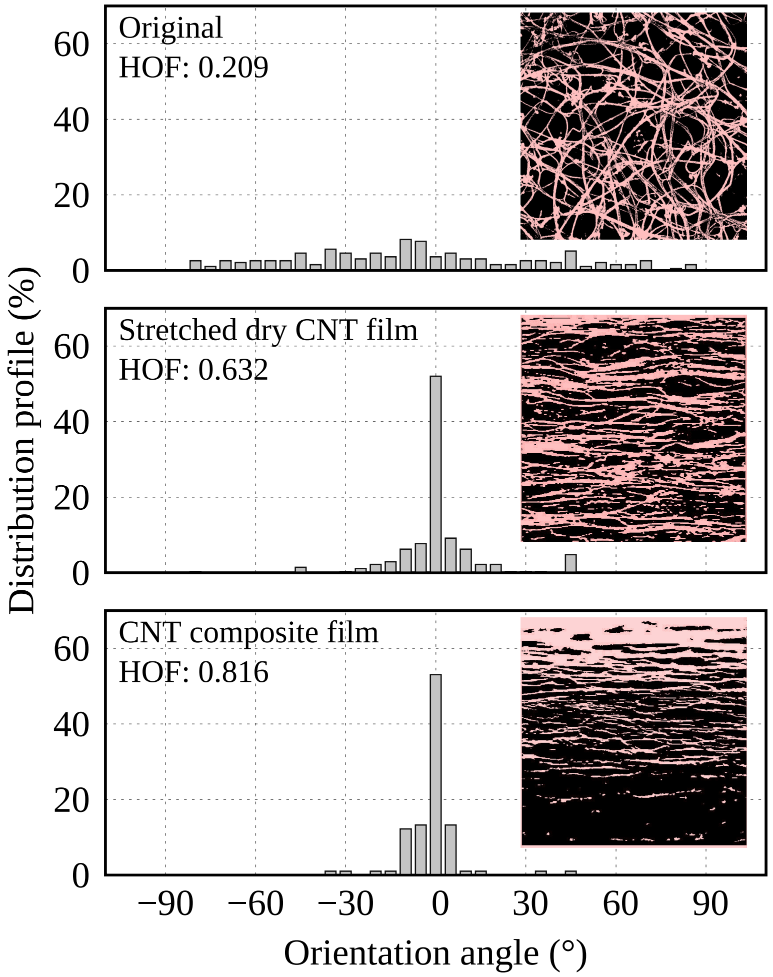

Figure S8: Intensity profiles of orientation angle for the as-produced CNT film, directly stretched CNT film, and the super-strong CNT/BMI composite film, whose SEM images are provided in Figure 2a, Figure S3b, and Figure 4c, respectively.

Here the intensity profile was obtained by using the '`Orientation`' property of '`regionprops`' in Matlab, where an SEM image should be first converted to be strongly contrasted.

Figure S8 shows the different intensity profiles for the as-produced CNT film, directly stretched CNT film, and the super-strong CNT/BMI composite film, respectively. The reference direction was taken to be the horizontal line for each image. For the original film, the orientation within [-5°,5°] was only 9.2% in the total counting of the orientation angles. After being stretched, the [-5°,5°] fraction increased up to 63.0% and 74.5%. However, as the stretching magnitude for the one-step stretching was only 20% in length, there were still some orientation angles larger than 40°

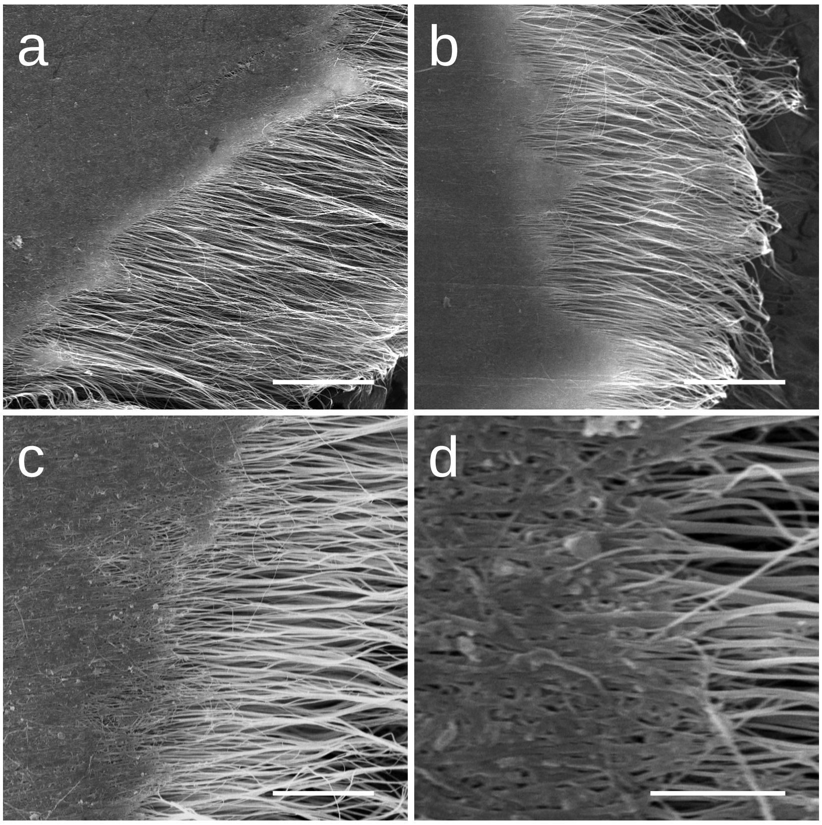

Figure S9: Fracture morphologies of CNT/BMI composite films. Scale bars are 10, 10, 5, and 1 µm, respectively.

(6.6% in the angle counting). According to these profiles, the HOFs were 0.209, 0.632, and 0.816, respectively. Obviously, the CNT alignment was significantly improved after the one-step and multi-step stretching treatments.

10. Fracture morphology

Figure S9 shows fracture morphologies of CNT/BMI composite films (mass ratio 7:3). Pull-out mechanism dominated at the fracture and the length of bare CNTs (more than 20 µm) can be used to estimate the tensile strength by $\sigma l/d$ (see the schematic shown in Figure S10a), σ, d, and l being the interfacial shear strength between CNT and BMI, diameter of CNT bundle, and bare length at fracture. Here we assumed that the CNT bundles were separated from each other by a distance close to the bundle size, and the space between them was filled by BMI polymers. By using $\sim$30 MPa, $d = 50$ nm, and $l = 20$ µm, the strength was estimated to be 12 GPa, in nice agreement with experimental measurements.

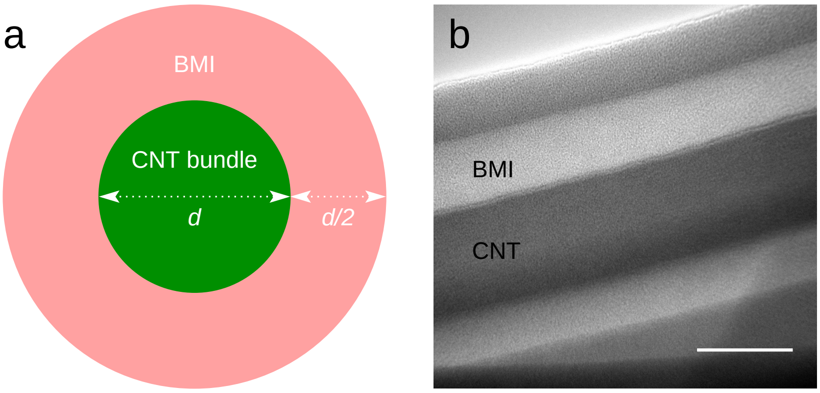

Figure S10: Schematic (a) and TEM characterization (b) of BMI-surrounded CNT bundles. (a) The bundle width is d and the BMI thickness is $d/2$. The shear contact area is proportional to πd and the total area is πd^2. (b) The real BMI thickness was in the same order with but slight smaller than the bundle size. Scale bar is 50 nm.

TEM characterization confirmed that the BMI thickness was in the same order of magnitude with the bundle size (Figure S10b). According to the strength estimation of $\sigma l/d$, to further improve the mechanical performance, the interfacial enhancement and reduction in bundle size will be the two major solutions.

Furthermore, from Figure S9d one can find that the CNT bundles were clearly unaggregated and the BMI polymers did not aggregate but uniformly distributed between the bundles.

11. Film thickness and specific tensile strength

The calculation of tensile strength requires the information of film width and thickness. The thickness of as-produced dry films was controlled to be within 10–30 μm. As the impregnated BMI resins occupied the pores in film, there was no thickness increase for the “wet” films. After being stretched, one-step or multi-step, the thickness decreased significantly with the stretching magnitude.

Figure S11 shows thicknesses of four stretched and cured composite films, with stretching magnitude ranged from ∼20% to ∼34% (from up to bottom). The final thicknesses was measured to be 5, 2.8, 2, and 1.7 μm, respectively. To avoid overestimation, we did not use the thickness of the thinnest segment to calculate the tensile strength, but the measurement on the thickest segment. For example, the thickness of the 34% stretched film was 2.5–3 μm.

As discussed in the main text, one can calculate the specific strength by measuring the fracture force and mass density of the CNT/BMI composite films. For the multi-step stretched films by

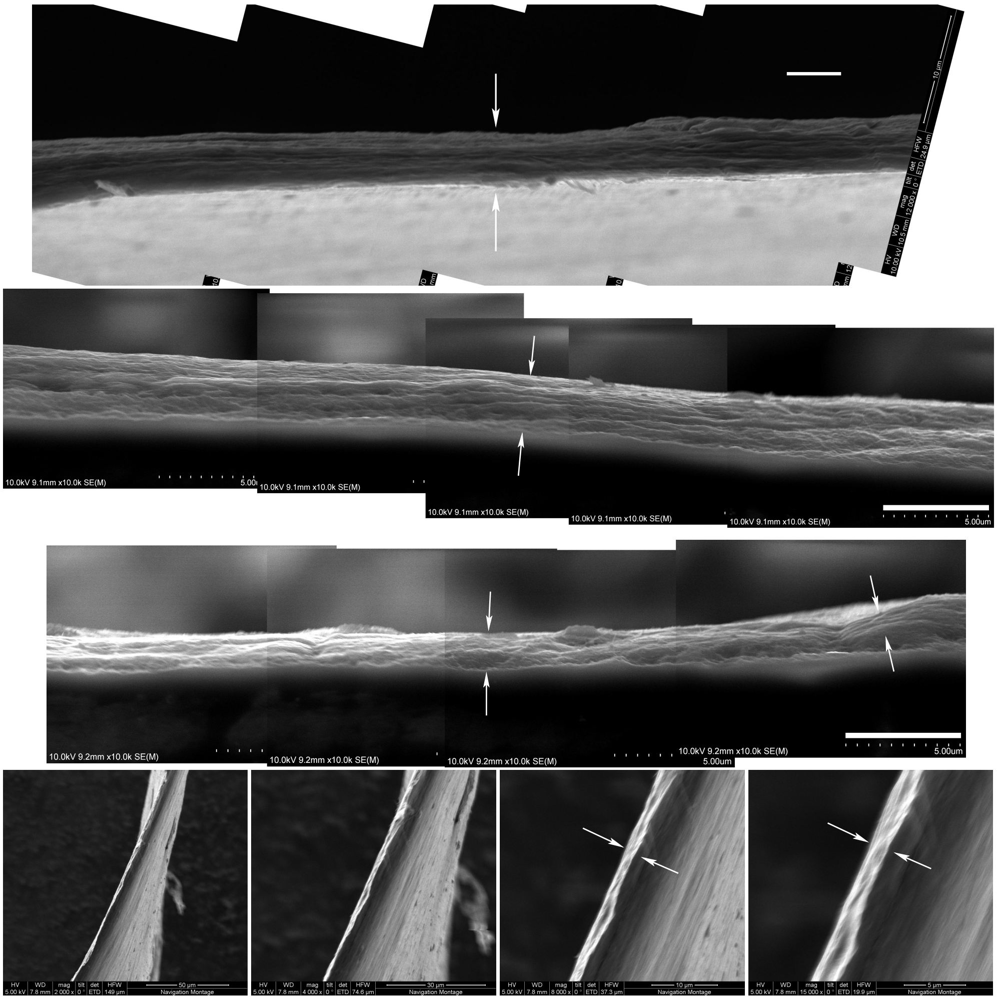

Figure S11: SEM characterization of film thickness. The unlabelled scale bars are all 5 μm.

$\sim$25%, the total mass for a 2 cm$\times$1 cm sample was 1.15 mg, corresponding to an area density of 0.58 mg/cm^2. The total force to fracture such film was about 16.5 N/mm in average (Figure S12), and thus the specific strength (by dividing the force per width by the area density) was $\sim$2.87 N/tex. When the stretching magnitude was improved to 34%, the fracture force per width was $\sim$19.5 N/mm, the area density decreased slightly to 0.46 mg/cm^2, and the specific strength was $\sim$4.24 N/tex. As the mass density was about 1.55 g/cc, the product of specific strength and volumetric density was 6.57 GPa. This means that all the measured data, including the force, film thickness, and mass density were completely consistent.

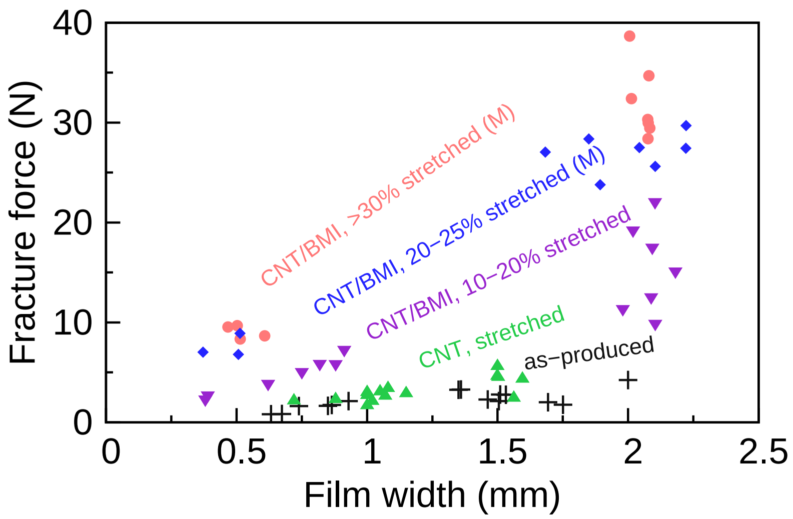

Figure S12: Fracture load as a function of sample width for different films.

12. Summary

We provide here detailed information of CNT growth, BMI resins, and impregnation and stretching techniques. Based on the unique properties of raw materials (entanglement, unaggregation, high plasticity, and low softening point) and the multi-step stretching method which was applied on resin-impregnated CNT films, we have been able to obtain a magic composite structure where neither CNTs nor polymers formed aggregated phases, a big step to approach the ideal composite structure to fully utilize all the CNT surfaces in load transferring. The highest tensile strength was 6.94 GPa (or 4.24 N/tex), much higher than the strength of carbon fiber reinforced polymers. The CNT/BMI composite films also exhibited high ability to conduct electricity.

References

[1] Y.-L. Li, I. A. Kinloch, A. H. Windle, *Science* **2004**, *304*, 276.

[2] Q. Cheng, J. Bao, J. Park, Z. Liang, C. Zhang, B. Wang, *Adv. Funct. Mater.* **2009**, *19*, 3219.

[3] Q. Cheng, B. Wang, C. Zhang, Z. Liang, *Small* **2010**, *6*, 763.

[4] S. Li, X. Zhang, J. Zhao, F. Meng, G. Xu, Z. Yong, J. Jia, Z. Zhang, Q. Li, *Compos. Sci. Technol.* **2012**, *72*, 1402.

[5] X. Wang, Z. Z. Yong, Q. W. Li, P. D. Bradford, W. Liu, D. S. Tucker, W. Cai, H. Wang, F. G. Yuan, Y. T. Zhu, *Mater. Res. Lett.* **2013**, *1*, 19.

[6] Li, Z., Yang, M., Huang, R., Zhang, M., and Feng, J. *J. Appl. Polym. Sci.* **80**(12), 2245–2250 (2001).

[7] Xu, M., Futaba, D. N., Yumura, M., and Hata, K. *ACS Nano* **6**(7), 5837–5844 (2012).
